# Establishment and Characterization of a New Cell Line of Canine Inflammatory Mammary Cancer: IPC-366

**DOI:** 10.1371/journal.pone.0122277

**Published:** 2015-03-25

**Authors:** Sara Caceres, Laura Peña, Paloma J. de Andres, Maria J. Illera, Mirtha S. Lopez, Wendy A. Woodward, James M. Reuben, Juan C. Illera

**Affiliations:** 1 Department of Animal Physiology, Surgery and Pathology, School of Veterinary Medicine, Complutense University of Madrid (UCM), Spain; 2 Department of Animal Medicine, Surgery and Pathology, School of Veterinary Medicine, Complutense University of Madrid (UCM), Spain; 3 Department of Radiation Oncology, The University of Texas MD Anderson Cancer Center, Houston, TX, United States of America; 4 Department of Hematopathology, The University of Texas MD Anderson Cancer Center, Houston, TX, United States of America; University of Alabama at Birmingham, UNITED STATES

## Abstract

Canine inflammatory mammary cancer (IMC) shares epidemiologic, histopathological and clinical characteristics with the disease in humans and has been proposed as a natural model for human inflammatory breast cancer (IBC). The aim of this study was to characterize a new cell line from IMC (IPC-366) for the comparative study of both IMC and IBC. Tumors cells from a female dog with clinical IMC were collected. The cells were grown under adherent conditions. The growth, cytological, ultrastructural and immunohistochemical (IHC) characteristics of IPC-366 were evaluated. Ten female Balb/SCID mice were inoculated with IPC-366 cells to assess their tumorigenicity and metastatic potential. Chromosome aberration test and Karyotype revealed the presence of structural aberration, numerical and neutral rearrangements, demonstrating a chromosomal instability. Microscopic examination of tumor revealed an epithelial morphology with marked anysocytosis. Cytological and histological examination of smears and ultrathin sections by electron microscopy revealed that IPC-366 is formed by highly malignant large round or polygonal cells characterized by marked atypia and prominent nucleoli and frequent multinucleated cells. Some cells had cytoplasmic empty spaces covered by cytoplasmic membrane resembling capillary endothelial cells, a phenomenon that has been related to s vasculogenic mimicry. IHC characterization of IPC-366 was basal-like: epithelial cells (AE1/AE3+, CK14+, vimentin+, actin-, p63-, ER-, PR-, HER-2, E-cadherin, overexpressed COX-2 and high Ki-67 proliferation index (87.15 %). At 2 weeks after inoculating the IPC-366 cells, a tumor mass was found in 100 % of mice. At 4 weeks metastases in lung and lymph nodes were found. Xenograph tumors maintained the original IHC characteristics of the female dog tumor. In summary, the cell line IPC-366 is a fast growing malignant triple negative cell line model of inflammatory mammary carcinoma that can be used for the comparative study of both IMC and IBC.

## Introduction

Inflammatory mammary cancer (IMC) is the most aggressive mammary neoplasia that affects female dogs [1, 2]. Its counterpart disease in humans is known as inflammatory breast cancer (IBC) and accounts for less than 6% of human breast cancer diagnoses with a poor survival in women [3, 4]. In both species, this type of cancer is highly angiogenic and angioinvasive [5–8]. The main histological characteristic of the disease in both species is the massive invasion of dermal lymphatic vessels by neoplastic cells which blocks lymph drainage causing the characteristic edema [1, 9].

There are considerable epidemiologic, clinical and histopathological similarities shared by IBC and IMC, therefore the latter is considered a good spontaneous model to study the human disease [2, 8]. The use of cell lines in cancer research offers advantages in the examination of cell growth and progression [10–12]. More than 33 human breast cancer cell lines from primary tumors, metastatic tumors and pleural effusion have been established [10, 11, 13]. However, for performing studies on IBC, the cell lines availability are limited to SUM149, SUM 190 and MDA-IBC-3, FC-IBC02 [12, 14, 15].

In the last years several canine mammary carcinoma cell lines have been developed [16–18] although, none of them is a canine inflammatory cell line. Thus, it is desirable to develop and establish a canine IMC cell line to compare the inflammatory breast cancer type in both species and to facilitate further in vitro studies. The aim of this study was to establish the first IMC cell line (IPC-366) and to characterize it in terms of immunophenotype and tumorigenicity.

## Materials and Methods

### Tumor specimen

**Fig 1:**
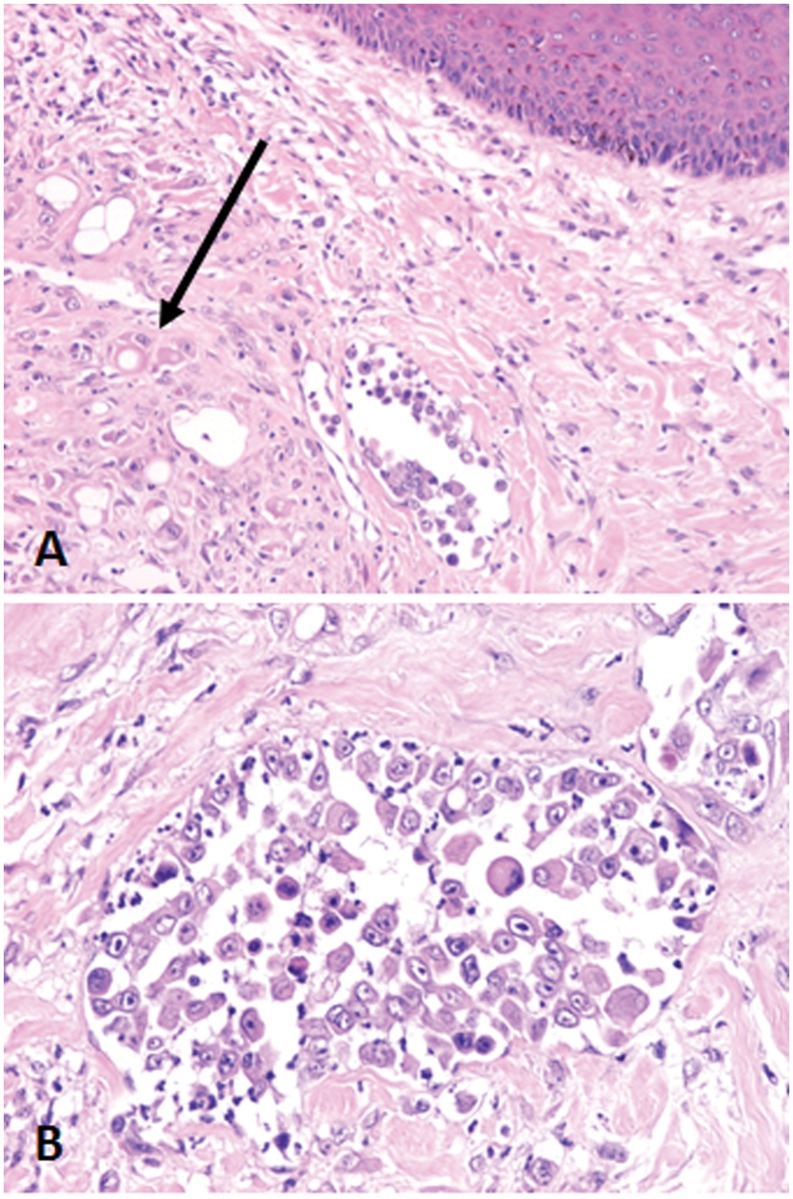
Primary canine inflammatory mammary carcinoma origin of the cell line IPC-366. Tumor paraffin sections, H&E. A (10X) y B (20X). Neoplastic emboli in superficial dermis. Tumor cells exhibit marked anisocitosis and anisokaryosis, and evident large nucleoli. Infiltrating tumor cells (arrow).

An IMC cell line (IPC-366) was established from a canine primary IMC sample obtained immediately after euthanasia of a female dog clinically and histopathologically diagnosed with IMC, according to the previously IMC diagnostic features published for this species [1, 19]: rapidly growing disease in the mammary glands and overlaying skin, with erythema, firmness, warmth, edema, thickening, and signs of pain, according to the referring veterinary clinician. The original canine mammary tumor was diagnosed as a solid carcinoma with multiple tumor emboli in superficial dermal lymphatic vessels (Fig. 1), confirming the diagnosis of IMC [2, 20]. After euthanasia, tumor samples were rapidly obtained at necropsy and processed for histopathological confirmation of IMC and cell culture. Tumor fragments (1.5 cm) were placed in Modified Eagle’s Medium (MEM) with penicillin-streptomycin solution (Sigma Aldrich, Madrid).

The histopathology and necropsy was performed at the Pathology Service of the Complutense Veterinary Clinical Hospital (Complutense University of Madrid). The Institutional Animal Care and Use Committee of the University Complutense of Madrid, Spain approved the clinical and experimental protocols for this study (number: 115). All procedures were completed in accordance with the Guide for the Care and Use of Laboratory Animals and conformed to the relevant EU Directive.

### Establishment of the cell line

Tumor tissue was fragmented and washed (3 times) with Hank’s Balanced Salt Solution with 1% of penicillin-streptomycin solution (Sigma Aldrich). Then, the tumor fragments were disaggregated with collagenase type 1A (Sigma Aldrich) at 37°C in a humidified atmosphere of 5% carbon dioxide for 2 hours with continuous agitation. Thereafter, the disaggregated tissue suspension was centrifuged at 1000 rpm, 20°C for 5 min and the pelleted cells resuspended in Dulbecco’s Modified Eagle Medium Nutrient Mixture F-12 Ham (DMEM/F12) containing 10% fetal bovine serum (FBS) (Sigma Aldrich), 1% penicillin-streptomycin solution and 1% L-glutamine (Sigma Aldrich). The cell suspension was placed in 25-cm^2^ culture flasks (Controltecnica Instruments, Madrid) and maintained in a humidified atmosphere of 5% carbon dioxide at 37°C. Cell culture was observed daily by a phase-contrast microscopy.

Once the cultured cells reached 90% confluence they were washed with Hank’s balanced salt solution supplemented with peninicilin-streptomicin solution and dispersed in 0.25% trypsin (Sigma Aldrich) with PBS containing EDTA. Subcultures were prepared from dispersed cells by reseeding in new 25 cm^2^ flasks at a concentration of 1x10^5^ cells/ml. Other dispersed cells were suspended in DMEM/F12 containing 20% FBS and 10% dimethylsulfoxide (DMSO) (Sigma Aldrich) and cryopreserved at -80°C. The establishment of IPC-366 was considered at passage 30^th^.

### Growth Assay

Cells at passage 30^th^ were seeded at 1xl0^5^ cells in 25-cm^2^ culture flasks and maintained in complete DMEM/F12 medium at 37°C for 7 days. Every 24 h, three replicative flasks were trypsinized and the cells were counted with a hemocytometer. A growth curve was established, and the cell growth doubling time was estimated from its exponential growth phase.

### Tumorigenicity assay

Given the well-appreciated pros of murine xenograft models, a suspension of 10^6^ IPC-366 cells was subcutaneously inoculated in the ventral region of ten 8-weeks-old female Balb/SCID mice. The mice were inspected weekly for the development of tumors. When tumors were detected, they were weekly monitored by palpation and measured by calipers. Mice were sacrificed after 30 days of inoculation, and tumors and organs were collected at necropsy and placed in 4% paraformaldehyde (pH = 7.4) for histological examination.

### Cytology, histopathology and immunohistochemistry

Cellular smears were prepared and stained using Diff-Quick for cytomorphological assessment. IPC-366 immunophenotyping was performed in canine original tumor and tumor pellets using the following markers (Table 1): pancytokeratin (AE1/AE3), cytokeratin 14 (CK14), p63, vimentin (vim), α-smooth muscle actin (sma), estrogen and progesterone receptors (ER and PR), Human Epidermal Receptor-2 (HER-2), cyclooxigenase-2 (COX-2), E-cadherin (E-Cad), and Ki-67 (proliferation marker). Paraffin sections were placed in a PT module (Lab Vision) containing an EDTA buffer solution (pH 8.0) (Master Diagnostica, MAD-004072R/D), heated for 20 minutes at 95°C and cooled down to 60°C. After this high temperature antigen retrieval protocol, slides were rinsed in warm tap water and placed in an automated Immunostainer device (Lab Vision Corporation, Fremont, CA) for immunohistochemistry using a peroxidase detection system (Table 1). After immunostaining the slides were counterstained with hematoxilin and permanently mounted with Depex. Corresponding negative control slides were processed by replacing the primary antibody with non-reactive antibody. Slides from human and canine mammary tumors with previously demonstrated reactivity to the primary antibody and tissue internal controls were used as positive controls.

**Table 1:** Technical data of specific antibodies and peroxidase-developing systems for immunohistochemistry.

Primary Antibody	Type, Clone*	Manufacturer**	Incubation	Detection system
**Pancytokeratin**	Mab AE1/AE3	Prediluted,MAD-001000QD	60min	UltraVision Quanto-HRP, MAD-021881QK
**Cytokeratin 14**	Mab LL02	AbD Serotec 1:1000 LL02	60 min	UltraVision Quanto-HRP, MAD-021881QK
**P63**	Mab 4A4	Prediluted, MAD-000479QD	30 min RT	UltraVision Quanto-HRP, MAD-021881QK
**Vimentin clone**	Mab SP20	Prediluted, MAD- 000326QD	20 min RT	UltraVision Quanto-HRP, MAD-021881QK
**Smooth muscle actin**	Mab 1A4	Prediluted, MAD- 001195QD	30 min RT	UltraVision Quanto-HRP, MAD-021881QK
**Estrogen Receptor**	Mab 1D5	DAKO 1/30 M7047	60 min RT	Dako EnVision- HRP K4007
**Progesterone Receptor**	Mab 1E2	Prediluted Ventana 790–2223	90 min RT	UltraVision Quanto-HRP, MAD-021881QK
**HER-2**	Pab	Dako A0485 1/1000	20 min RT	Dako EnVision-HRP K5007
**COX-2**	Mab SP21	Prediluted, MAD- 000335QD	150 min RT	UltraVision Quanto-HRP, MAD-021881QK
**E-Cadherin**	Mab EP700Y	Prediluted, MAD-000051QD	60 min RT	UltraVision Quanto-HRP, MAD-021881QK
**Ki-67**	Mab SP6	Prediluted, MAD 000310QD	90 min	UltraVision Quanto-HRP, MAD-021881QK

* Mab = monoclonal antibody, Pab = polyclonal antibody.

** MAD = Master Diagnostica.

The primary canine mammary tumor, the cell line and xenotransplanted tumors were considered positive for AE1/AE3, CK-14, p63, vimentin, actin, E-Cad and COX-2 when more than 10% of the neoplastic cells were positive [21]. Beside this, IPC-366 phenotype markers were scored in pellet sections by a computer assisted image analyzer (image J), counting up to 1000 cells. For HER-2 evaluation, 3+ positive scoring following the recommended guidelines of ASCO (American Society of Cancer Oncology, Her-2 3+) was considered. Ki-67 proliferation index (PI) was determined by counting Ki-67 positive and negative nuclei in 1000 neoplastic cells. Every immunostained nucleus was considered positive regardless of the intensity. The PI, or proportion of positive neoplastic cells in each sample, was calculated [22].

### Electron microscopy

IPC-366 cells were harvested and fixed with 2.5% glutaraldehyde (Panreac, Barcelona, Spain) and 4% paraformaldehyde (Panreac) solution. Then the cells were incubated with 0.1 M cacodylate buffer at 4°C overnight. The fixed cells were treated with 2% osmium tetroxide (Panreac) and 3% ferrocyanide (Panreac) solution (diluted in PBS) for 1 h. They were then washed in distilled water and dehydrated in acetones of increasing percentage (30, 50, 70, 80, and 100%). The samples were gradually infiltrated in a Müllenhauer mixture resin (Lowicryl resin, Sigma Aldrich), and solidified at 60 8°C for 24 h. The embedded cells were sectioned at the National Electron Microscopy Center (Madrid). Images were obtained using a JEOL JEM 3000F transmission electron microscope.

### Cytogenetic analyses

Chromosome preparation for aberration Chromosome (AC) and G banding, were obtained from short term cell culture with Dulbecco's Modified Eagle Medium, DMEM-F12 (Lonza, USA) containing 10% fetal bovine serum (FBS) (Atlanta Biologicals, USA), 1% penicillin-streptomycin solution and 1% L-glutamine (Gibco- life Technology, USA). The cell suspension was placed in 75 cm^2^ culture flasks (Corning, USA) and was maintained in a humidified atmosphere of 5% carbon dioxide at 37°C for 54h. Colcemid (Gibco- Life Thecnology,USA) was added for 2h before the cell suspension was harvested. The preparation followed methods for hypotonic treatment (KCL) (Sigma Aldrich, USA) and fixation with 3:1 methanol:glacial acetic acid (Sigma Aldrich, USA). Slides were allowed to age before the staining with Giemsa and G banding [23]. The results were processed with Applied Spectral Imaging, Version 6.0 for the review of the metaphases and Karyotyping.

## Results

### Microscopic morphology of IPC-366 and growth assay

**Fig 2:**
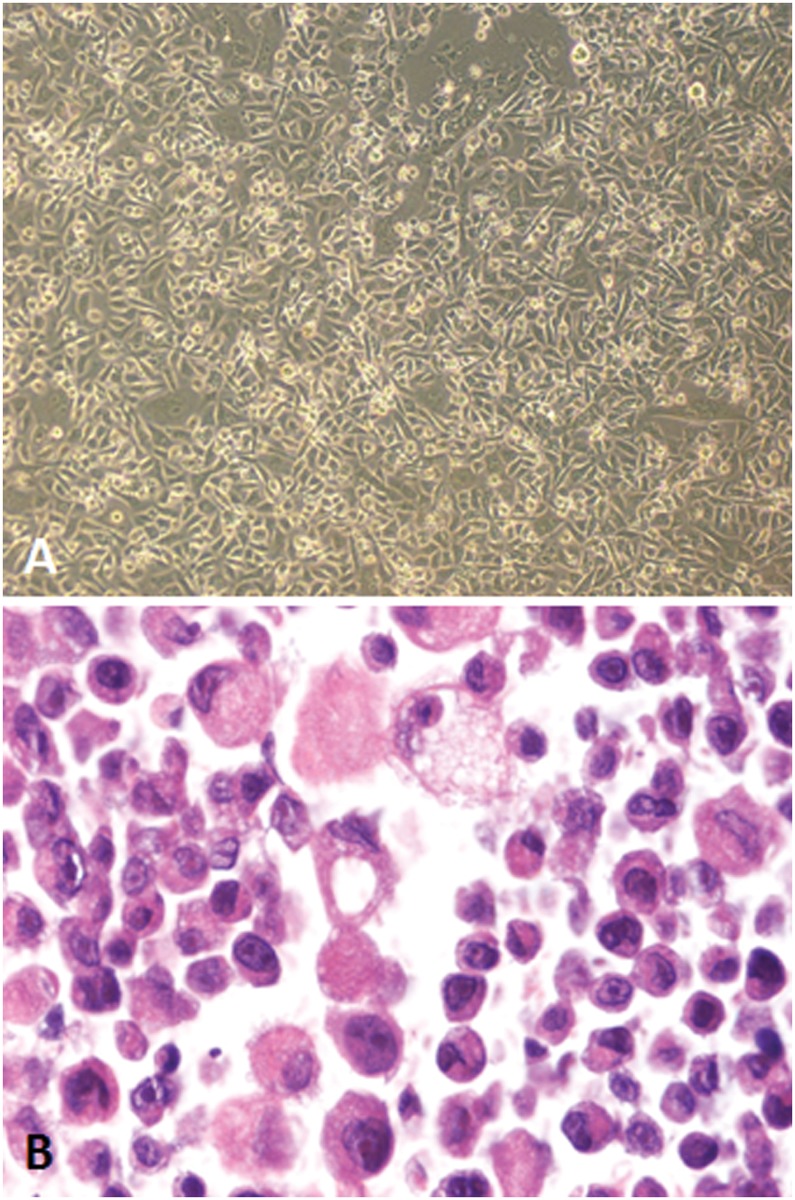
Morphology of IPC-366 cells. A) IPC-366 cells in culture at inverted microscopy (40X). B) IPC-366 pellet; paraffin section, H&E (40X). Highly malignant tumor cells with epithelial morphology; marked anisocytosis and anisokaryosis and evident nucleoli. Endothelial-like cell (ELC) showing cytoplasmic clear space (arrow). IPC-366 ultrastructural features were evaluated by electron microscopy. The cells had abundant cytoplasmic projections, numerous organelles and proteinaceous secretory products. By electron microscopy, the clear cytoplasmic vacuoles seen by optic microscopy, resulted empty spaces lined by cytoplasmic membranes that occasionally joined to form an internal lumen (ELCs, vasculogenic mimicry). (Fig. 3C-H).

**Fig 3:**
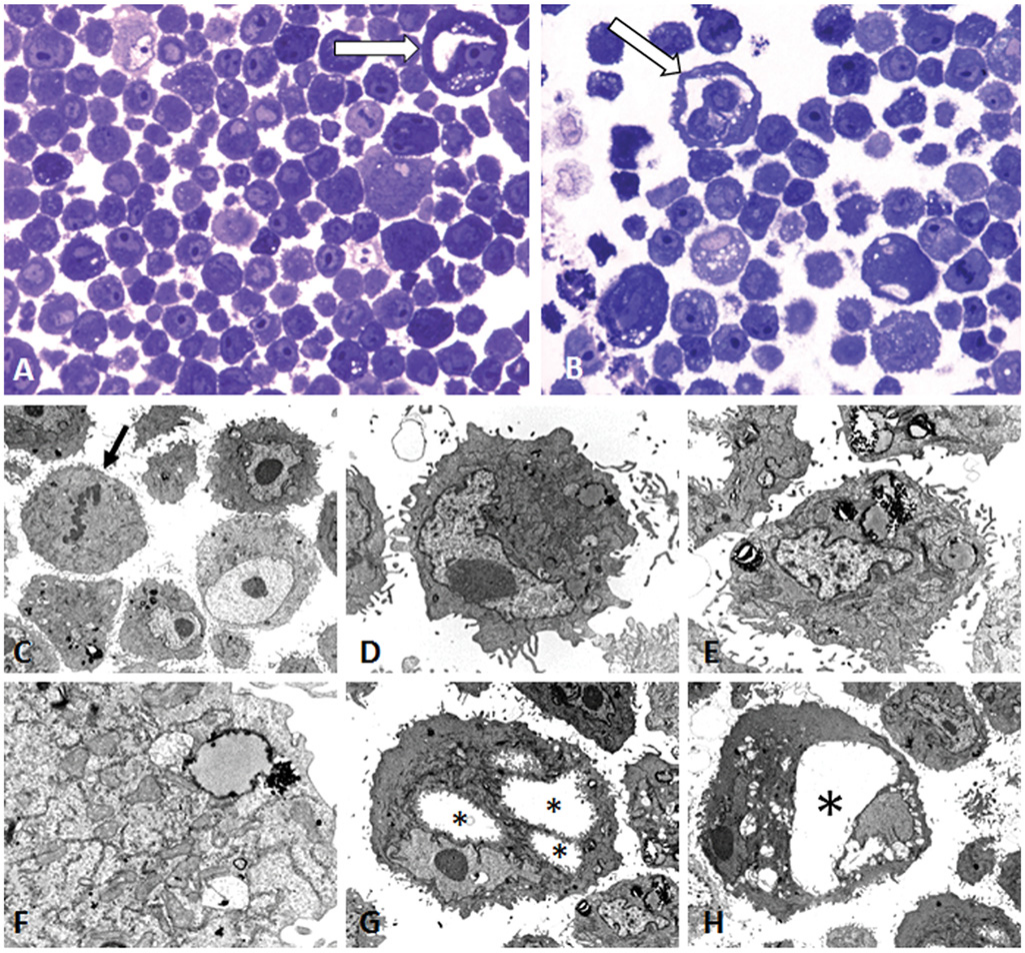
IPC-366 cells in pellet resin sections. A and B) Semithin sections stained with toluidin blue (40X); some endothelial-like cells are observed (arrows). C to H) Ultraestructure in ultrathin sections. C) Cells with large nuclei and evident nucleoli. Mitosis (arrow). D) Single cell with elongated nucleus, very evident nucleolus and numerous organelles, vacuoles and proteinaceous secretion. E) Tumor degenerated cell with shrunken nucleus and abundant proteinaceous secretion. F) Cytoplasm of a tumor cell containing numerous organelles (mitochondria, endoplasmic reticulum) and a secretory vacuole. G) Tumor cell with three cytoplasmic empty spaces (asterisks) covered by cytoplasmic membrane; progression to a cytoplasmic “lumen” characteristic of endothelial-like cells. Evident nucleolus. H) Binucleate tumor cell with a cytoplasmic central empty space (“lumen”, asterisk) (ELC) resembling a capillary vessel (vasculogenic mimicry). Figs. E and G show different fields from the same grid. At 25^th^ passage, cells were cryogenic storage (-180°C). Re-cultured thawed cells presented similar growth and morphological characteristics with a viability of 95–99%. The doubling time of IPC-366 cells at 30^th^ passage was approximately 24 hours and the cells reached a plateau on day 6 (Fig. 4).

IPC-366 cells adhered to culture flaks presented epithelium-like morphology and characteristics (Fig. 2A). Histological observation of IPC-366 cells in smears and H&E stained paraffin sections from pellets revealed large or very large round cells (mean diameter = 18.01μm, minimum = 7.35μm—maximum 583.33μm). Cytoplasms were slightly basophilic and frequently contained basophilic aggregates. The nuclei were very large (minimum = 5.25 μm—maximum = 13.38 μm) with evident large and numerous nucleoli (minimum = 3.74 μm—maximum = and 8.24 μm). Anisocytosis and anisokaryosis were marked. Frequently, IPC-366 cells showed multiple coalescent cytoplasmic clear vacuoles and elongated eccentric nucleus. Some large cells presented morphological features of endothelial cells: a rim of elongated encircled cytoplasm containing elongated eccentric nuclei (endothelial-like cells, ELCs, suggestive of vasculogenic mimicry) (Figs. 2B, 3A and 3B). Highly malignant multinucleated cells were frequently seen (8.72% of multinucleated ce4lls in a 20X microscopic field).

**Fig 4:**
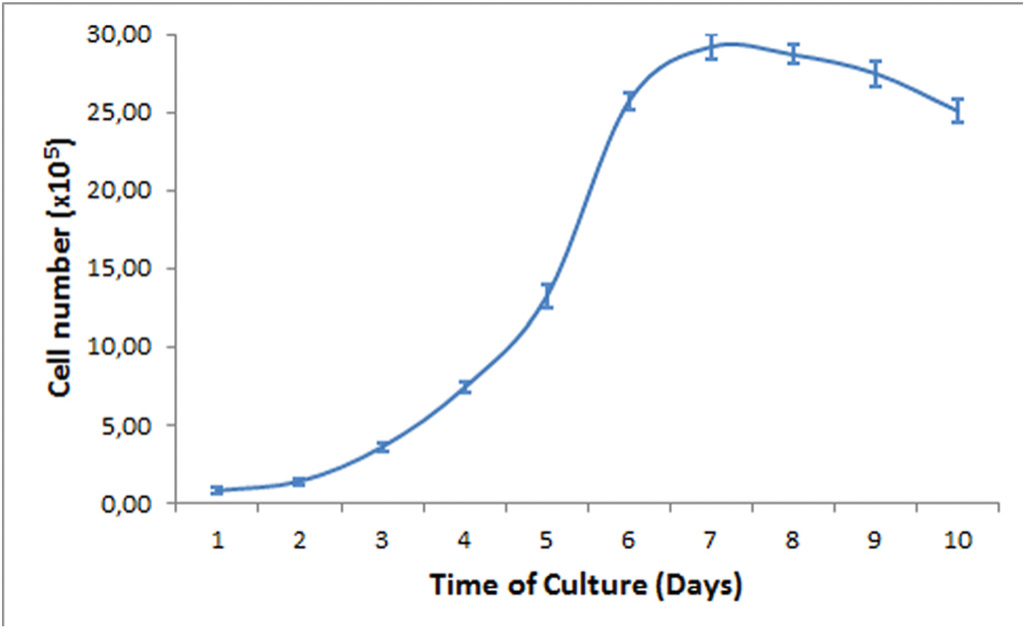
Growth curve of IPC-366 at 30th passage.

### Tumorigenicity

**Fig 5:**
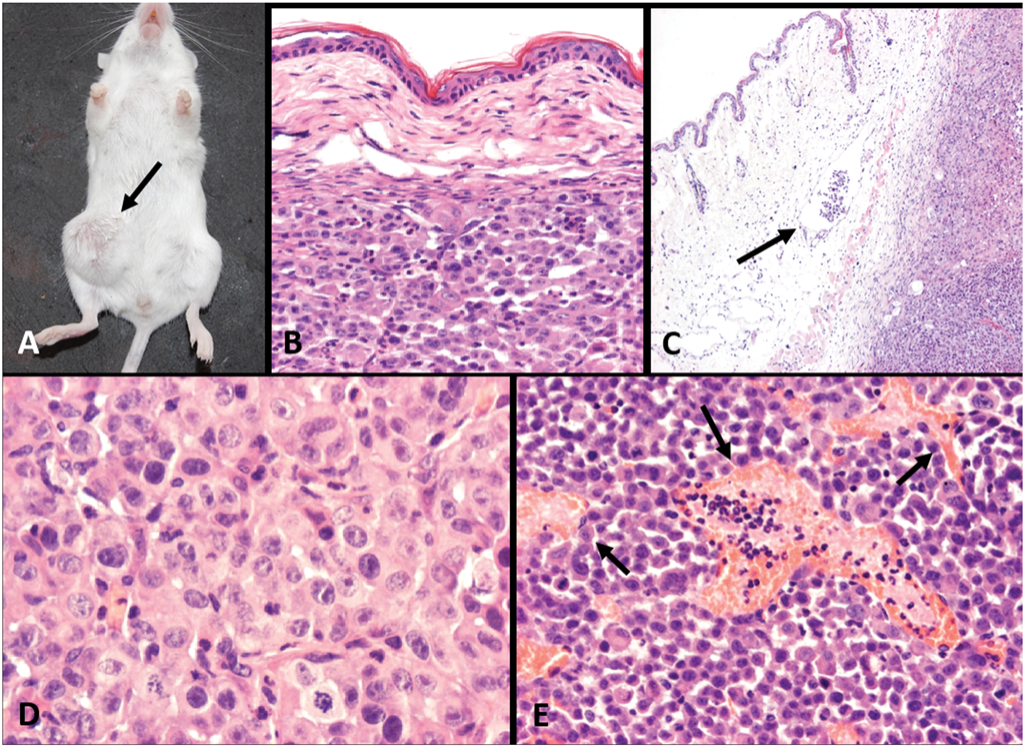
Tumors from mice inoculated with IPC-366. A) IPC-366 mouse xenografts at four weeks (arrow). B to E: tumor paraffin sections H&E. B) Solid tumor infiltrating the dermis (10X). C) Neoplastic embolus in a superficial dermal lymphatic vessel (arrow) and solid tumor; marked edema in dermis (4X). D) Highly malignant tumor cells with marked anisocytosis and anisokaryosis; evident nucleoli (40X).E) Microvascular channels lined up by neoplastic cells (vasculogenic mimicry, arrows) (20X).

Injected IPC-366 cells into the ventral side of female Balb/SCID mice originate tumors after 2 weeks of inoculation (100% of inoculated mice, n = 10, length: 0.6 and 0.9 cm^3^, width: 0.2 and 0.4 cm^3^). Two weeks after the observation of the xenotransplanted tumors their size was up to 1.5 cm^3^ and mice were euthanized (length: between 1.3 and 1.7 cm^3^, and width: between 0.8 and 1.2) (Fig. 5A). The histological examination revealed a highly infiltrating, poorly demarcated, unencapsulated densely cellular neoplastic growth, extending to the dermis and striated muscle. Neoplastic cells arranged in solid masses with scant stromal and had similar morphology and malignancy to that described for IPC-·66. Mitotic index was very high and atypical mitoses were frequently observed. (Fig. 5B-C). The presence of emboli on dermis capillaries (Fig. 5D) confirmed the histological characteristic of inflammatory mammary carcinoma. The presence of vasculogenic mimicry (highly malignant tumor cells resembling endothelial cells and surrounding vascular spaces (vasculogenic mimicry) (Fig. 5E), was a frequent finding.

### Immunohistochemical characterization of IPC-366 phenotype

**Fig 6:**
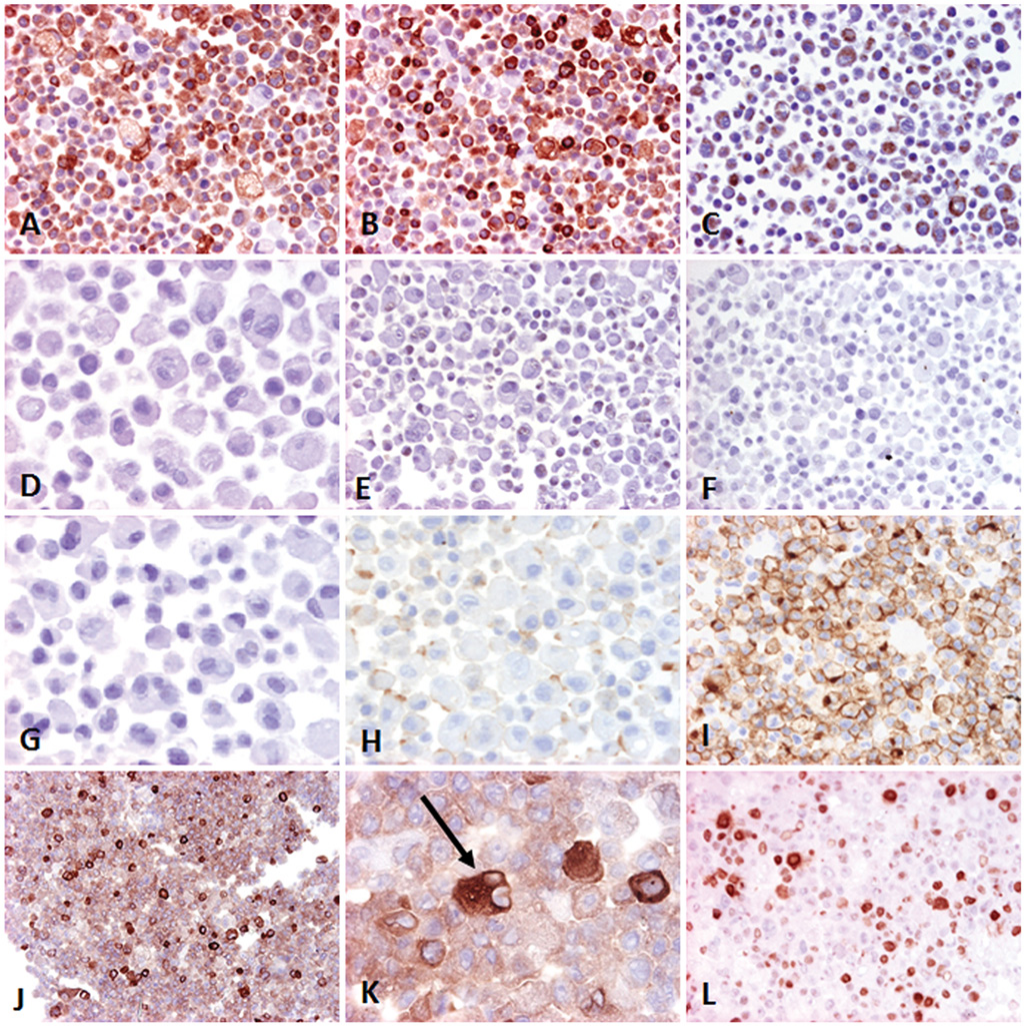
Immunohistochemistry of IPC-366 pellet, paraffin sections. A) Pancytokeratin (AE1/AE3) (10X); intense immunolabeling and numerous positive cells. B) CK14; intense immunolabeling and numerous positive cells (10X). C) Vimentin; moderate immunolabeling and numerous positive cells (10X). D) p63; negative (20X). E) α-smooth muscle actin, negative cells (10X). F) Estrogen receptor; negative cells (20X). G) Progesterone receptor; negative cells (20X). H) HER-2; negative cells (20X). I) E-Cadherin; intense membranous immunolabeling in numerous cells (20X). J) COX-2; most of the cells are moderately positive; some cells are intensely positive (10X). K) COX-2; some cells strongly positive showing features of malignancy (binucleated cell, arrow) (40X). L) Ki-67 proliferation marker; many nuclei are positive (10X).

Primary canine inflammatory mammary carcinoma and IPC-366 cells presented similar immunoreaction to the immunophenotype markers used. They were positive to a general epithelial cell marker (pancytokeratin, AE1/AE3), and a basal cell marker (CK14), with a 95.11% and 92.58% of positive cells in the pellet, respectively (Fig. 6A-B). All samples had also a positive reaction to vimentin, showing the IPC-366 an 81.14% of positive cells in the pellets. (Fig. 6C). Myoepithelial cell markers p63 and actin were negative (Fig. 6D-E), as well as hormonal receptors (ER, PR), and HER-2. (Fig. 6F-H). More than 90% of IPC-366 overexpressed E-Cad (Fig. 6I) and COX-2 (Fig. 6J-K). COX-2 immunolabelling was very strong in 10% of cells. All samples had a high proliferation rate measured with Ki-67. The mean Ki-67 proliferation index of IPC-366 in pellets was 87.15% (Fig. 6L).

### Cytogenetic analyses

The analysis was made after two cell cycles, The cells were divided for chromosome aberration (AC) test and Karyotype. Four hundred cells were examined and 69% of the cells showed the normal karyotype (2n = 78). On the other hand, metaphases examined detected at least one or more abnormalities in 31% of the cells (Table 2). The autosomes chromosomes have similar morphology, the number and all of them are Acrocentric, and the chromosome X metacentric. 2% of the observed cells have numerical aberration involving chromosomes X, 12 and 17 using the ideogram for the G banding analysis in Dog [24, 25]. The AC test reveals that 30% of the cell viewed have breaks: 15% chromatic, 8% chromosomic aberration and 6% gaps.

**Table 2:** Aberration found in the Karyotype.

Break point	dm	Gain	Deletion	Inversion
X (23)	12	17 (12–14)	del 12 (21–23)	inv 9 (12:14)
17 (21)	11	34 (221–23)	del 16 (21–24)	inv 17 (11:14)
11 (13)	16	18 (221–23)	del 11 (12–16)	inv 2 (12:23)
15 (15)	5	16 (12–14)	del 26 (21–23)	inv 3 (22:34)
26 (13)	9	9 (11–12)	del 22 (11–12)	
27 (13)	6	8 (11–12)	del 5 (21–33)	
5 (34)		3 (11–12)	del 20 (14–16)	
20 (12)		1 (11–12)	del 25 (21–24)	
9 (22)		24 (11–12)	del 17 (12–16)	
		13 (11–12)	del 19 (22–24)	
			del 26 (21–23)	
			del X (21–26)	

## Discussion

IMC and IBC are a subtype of aggressive mammary cancer that affects female dogs and humans, respectively [1, 2, 26]. *In vitro* studies using established cancer cell lines offer knowledge about the progression and growth of breast cancer in general and IBC in particular [11, 12]. There are few models available for the study of the biology of IBC [10, 12, 15]. Most studies have been carried out using cell lines such as SUM190 and SUM149. SUM149 is a triple negative cell line whereas SUM190 is ER/PR negative and HER-2 positive. Other less studied IBC cell lines are: MDA-IBC3, KPL4 and WIBC-9 (all ER/PR negative and HER-2 positive). In the past year, it has been described and characterized the new FC-IBC02 cell line [15]. Canine IMC shares many clinical, biological and pathological characteristics with the disease in humans, therefore it has been proposed as a good spontaneous animal model for the study the human disease [2]. Nevertheless, to the best of our knowledge, IPC-366 is the first canine IMC cell line established.

IPC-366 cell line reproduced the histological features of the canine primary tumor. It is a very aggressive, highly malignant cell line that inoculated to Balb/SCID mice can originate tumors that reproduce the histological features of the primary canine IMC: a very infiltrative tumor composed by anaplastic cells with lymphatic invasion of the superficial dermal lymphatics, being this latter characteristic the histological hallmark for both IBC and IMC histological diagnoses [2, 5, 26]. IPC-366 also preserved the original canine mammary IMC immunophenotype. According to the immunohistochemical results, IPC-366 is positive for panCK, CK14 and vimentin, and negative for actin, p63, ER, PR, HER-2, indicating that this cell line is a basal epithelial cell line.

Multimodal treatment for patients with IBC includes chemotherapy, followed by aggressive radiotherapy and surgery. As in other non-IBC breast cancers, anti-HER-2 or anti-hormonal targeted therapy are used in positive patients to these molecules [4]. In spite of this, survival of patients with IBC is very short [4]. Survival is even shorter in triple negative IBC cases (TN IBC, negative tumors to ER, PR and HER-2) that are especially resistant to the therapies [27].

Triple negative breast cancer (TNBC) accounts for approximately 10–15% of breast cancers and has a poorer prognosis than positive hormone receptor patients [28–30]. This type of breast cancer also an outcome characteristic of aggressiveness, invasiveness, early metastases and unresponsive to therapeutic targets [28], and is treated mostly with chemotherapy [30].

In vitro research on TN-IBC is conducted mainly on the triple negative cell line SUM149. This cell line has been used to identify genetic factors of the IBC phenotype [10]. Immunohistochemical results revealed that IPC-366 lacked ER, PR and HER-2 expression, being another novel triple negative cell line that can be used for the research of novel therapeutic targets.

The cell line IPC-366 is presented as a basal epithelial cell line with mesenchymal characteristics: positive immunoexpression to pancytokeratins (AE1/AE3) (general epithelial cell marker), cytokeratin 14 (for basal epithelial cells) and vimentin (general mesenchymal marker). The co-expression of cytokeratins and vimentin has been described in breast tumors associated to malignancy [31, 32]. Vimentin expression is also associated with an increase of malignancy of the cells in culture [31]. Thus, immunoexpression profile revealed that IPC-366 comprised in a basal-like subtype cell line with epithelial-mesenchymal transition (EMT) [33]. The basal-like subtype is characterized by a negative expression of ER, PR and HER2 completed with a CK14 and vimentin positive [28]. SUM149, a well-established IBC cell line, is also of basal like subtype [12]. The fact that canine IPC-366 cell line shares phenotypic characteristics with the human SUM149 cell line will allow comparative pre-clinical studies in a future. Negative immunoexpresion to the myoepithelial cell markers p63 and actin indicates that IPC-366 is not of myoepithelial origin. In contrast with human breast cancer, canine mammary cancer has frequent myoepithelial proliferations leading to complex and mixed tumors [20]. The absence of a myoepithelial cell lineage in IPC-366 will further facilitate proper comparative studies. These results demonstrated the epithelial origin of IPC-366 and its acquisition of mesenchymal characteristics as well as the absence of myoepithelial cells in this cell line. Thus, IPC-366 cells are an example of epithelial-mesenchymal transition. EMT is a process implicated in the tumor progression leading epithelial cells turning into mesenchymal cells [34, 35]. The acquisition of the mesenchymal phenotype involved the loss in epithelial markers expression as well as gain cell motility and tumor invasion increased [36]. IPC-366 overexpressed COX-2 in 90% of the cells. Recently, the overexpression of COX-2 has been related to EMT and invasiveness in breast cancer cell lines [37].

E-cadherin overexpression in IPC-366 has been also demonstrated. E-cadherin is a cell-cell adhesion protein in epithelial tissues encoded by the tumor suppressor gene CDH1. It is known that mutations in CDH1 increased proliferation and tumor invasion and metastasis [38–40]. Thus, the loss of function of E-cadherin plays a very important role in EMT and tumor survival [40, 41]. It is known that in IBC E-cadherin is overexpressed and localized around the tumor cell membrane, which accounts for the formation of lymphovascular embolus [42, 43].

IPC-366 is also characterized by a high proliferation measured by a growth assay and a Ki-67 index (87.15%), which ensures in vitro studies. Ki-67 expression has been associated with poor prognosis in malignant mammary tumors both in women and dogs [22, 44]. IPC-366 had higher proliferation index than the original tumor, suggesting an increase of proliferative capacity, probably by selection of the most malignant cells. The cytogenetic analysis shows the presence of structural aberration, numerical and neutral rearrangements, demonstrating a chromosomal instability. These data can be correlated with the results in patients with inflammatory breast cancer and using more sensitive and specific methods such as SKY, CGH Southern blot analysis and studies of expression pattern with Cdna microarray chips would increase the knowledge regarding genetic aberration and conserved segments who can open the window to found genetic markers. Histological features of malignancy in the primary canine mammary tumor and the cell line were similar with marked anisokaryosis and anisocytosis. The typical morphology of endothelial-like cells, which has been related to the vasculogenic mimicry (VM) phenomenon [7, 8] was observed similarly in the primary tumor and the derived cell line. The presence of ELCs should be considered as an interesting initial result that deserves further specific research. In the derived mice tumors, besides the observation of ELCs, it was possible to find large channels of VM. The higher proportion of highly malignant multinucleated cells found in IPC-366 and in the transplanted tumor in mice could represent also early stages of VM in highly undifferentiated ELCs, although further studies are needed to confirm this affirmation. This phenomenon defined as vasculogenic mimicry was first described in human melanoma and [45] consists in the generation of vascular channels by malignant tumor cells that acquire endothelial characteristics and provides de blood supply required for tumor growth and metastasis. The generation of functional vascular channels by the tumor is a marker of malignancy tumor cell phenotype [45, 46]. Vasculogenic mimicry does not appear in all types of cancer, but it frequently occurs in IBC [47] and IMC [7]. Recently, it was also identified in xenografts from IMC [48].

Ninety percent of IPC-366 cells were positive for COX-2. Some cells, including ELCs, were intensely positive for COX-2. COX-2 has a role in the invasive and angiogenic phenotype of IBC [49] and it has been related with lymphangiogenic pathway stimulation in IMC [8]. The relationship of COX-2 with tumor extravasation, tumor cell survival and distant metastasis re-initiation has been described [50]. COX-2 has been indicated to be a marker for ELCs involved in VM [7, 8, 51]. Although more studies are necessary to characterize the angiogenic characteristics of IPC-366, this cell line could represent a very interesting model for the study of VM and other angiogenic properties of IMC.

A role for COX-2 as a stem cell marker has been postulated recently [52]. The stem cell properties of IPC-366, under investigation, are not the purpose of the present study, but the high proportion of COX-2 positive cells and the presence of numerous ELCs seem to indicate that the rate of stem cells could be high. Complete future studies on stem cell phenotype will dilucidate this aspect of the IPC-366 cell line.

Fast tumorigenicity is another very important characteristic of IPC-366. This cell line is able to reproduce tumors *in vivo* very rapidly, without losing its original characteristics, which permit to study neoplastic growth in a biological animal environment. Although it is a much appreciated characteristic, not all the established cell lines are capable to reproduce tumors *in vivo*. MCF7 does not grow in mice, although other tumorigenic IBC cell lines such as SUM149 or SUM190 produce tumors in SCID mice xenografts after 6–8 weeks of injection of cells into mammary fat pad [53]. Xenografts from IPC-366 in Balb/SCID mice grew in 3 weeks with a frequency of 100%, showing that this cell line had a faster growth *in vivo*. IBC and IMC have a strong tendency to metastasize and invade lymphatic vessels [1, 5, 42]. IPC-366 xenografts in mice showed spontaneous metastases in lung and lymph nodes with a frequency of 100% (all injected animals suffer metastases). Therefore, IPC-366 could be considered a good model for the study of the metastatic mechanisms *in vitro* and *in vivo*. There are no IBC or breast cancer cell lines with this high capacity of metastases in the corresponding xenotransplanted mice; as an example, SUM149 produce metastases in lungs with a frequency of 2/3 [53].

In the last years, several canine mammary cancer cells lines have been established: DTK-E and DTK-SME [17], CMT1, CMT-U27, CMT9, P114, CNMp and CIPp [54], KTOSA5 and CSKOS [55], CHMp [56], CMT-W1 and CMT-W2 [16]. The reported tumorigenicity of the other canine mammary cancer cell lines indicate the tumor formation between 6 to 8 weeks after inoculation in SCID mice. The faster growth *in vivo* of IPC-366 cell line (4 weeks) represents an advantage compared with the other canine mammary cancer cell lines. IPC-366 is the first canine inflammatory mammary cancer cell line established. After inoculation in Balb/SCID mice, IPC-366 originated tumors that reproduced the characteristic tumor invasion of superficial dermal vessels, indicating that IPC-366 could be also used as a model of canine inflammatory mammary cancer *in vivo*.

Tumor cell lines are useful for enhancing our understanding of cancer development and treatment. We have established the first IMC cell line (IPC-366). IPC-366 is a new IMC triple negative epithelial basal cell line, highly malignant, that exhibits fast proliferation *in vitro* and fast growth (3 weeks) and distant metastases *in vivo*, that reproduces histological characteristics of IMC *in vivo*, and possess properties of VM *in vitro* and *in vitro*.

According to its malignancy, tumorigenicity and tumor marker profile, this new established IMC cell line IPC-366 should become a useful cell model not only for basic tumor biology studies but also for the development of potential therapeutic agents and for future interspecies comparative new therapeutical strategies against IMC/IBC.
